# The role of QI collaboratives in neonatology

**DOI:** 10.1038/s41372-024-02124-w

**Published:** 2024-10-09

**Authors:** Roopali Bapat, Stephen Pearlman

**Affiliations:** 1 Nationwide Children’s Hospital, Columbus, USA; 2 The Ohio State University, Columbus, Ohio USA; 3 ChristianaCare, Newark, DE USA; 4 Sidney Kimmel College of Medicine of Thomas Jefferson University, Philadelphia, PA USA

**Keywords:** Health care, Scientific community

## Abstract

Quality improvement collaboratives (QICs) use their collective experiences from participating centers to accelerate the translation of evidence into practice, resulting in reduced variation and improved clinical outcomes. There are several regional, national, and international QICs in neonatology. In this review, we discuss the framework and evaluate national QICs primarily based in US and share the contributions of selected studies. We found that the QICs in neonatology play a significant role in identification of target topics, developing best practices, improving provider knowledge, building QI capacity, and improving outcomes. The key strengths of QICs are that they produce more generalizable learnings, involve a larger patient population which enhances statistical analysis, and offer resources to smaller institutions. Limitations include institutions contributing unequally to the overall results, difficulty in interpreting results when multiple improvement strategies are applied simultaneously, and the possible lack of academic recognition for individual center leadership.

## Introduction

### What is a QI collaborative?

A quality improvement collaborative (QIC) is defined as a structured framework that amalgamates groups of practitioners from different healthcare organizations to improve a specifically identified aspect of the quality of their service [1–3]. QICs facilitate multidisciplinary teams to implement evidence-based knowledge by learning from one another, collaborating, testing changes, and utilizing data driven changes. Using their collective experiences, QICs can accelerate the translation of evidence into practice, resulting in reduced unnecessary variation and improved clinical care and outcomes [4–6]. A QIC usually consists of policy leaders and health stakeholders working toward a common goal of improving performance on a well-defined quality measure or set of measures related to a safety issue or clinical outcome [7]. A QIC takes a multifaceted approach to quality improvement (QI) that involves five essential features- a target topic, clinical and quality improvement experts, multi-professional teams from multiple centers, a model for improvement, and a series of structured activities geared towards improvement [8]. Multidisciplinary teams include key stakeholders at the individual centers and across the centers, including patients and families [6, 9, 10].

### Do QI collaboratives work?

QICs have reported significant improvements in targeted clinical processes and outcomes as described in a systematic review by Wells et al. [3]. Zamboni et al explored the relationship of QIC outcomes with context domains (health facility setting, project-specific factors, organizational and external factors) and mechanisms (intra- and inter- organizational changes). They reported that participation in a QIC improves knowledge, problem-solving skills, attitude, teamwork, shared leadership, and generates opportunities for capacity building [9]. They also identified that QIC outcomes are successful when the collaborative goals are aligned with national patient safety goals. Two other systematic reviews of QICs in healthcare have identified improvements in patient care and organizational performance [11, 12].

Success of a QIC depends on five general factors: the team’s ability to work together; their ability to learn and apply quality methods; the strategic importance of their work to their home organization; the culture of their home organization; and the type and degree of support from management [1]. Collective learning and an evidence-based approach help the collaborative models accelerate learning and process change amongst participating hospitals [2, 13].

However, QIC reports in adult literature have described limitations in design and methodologies for any conclusive evidence and the effects cannot be predicted with certainty [11, 12]. Often, it is difficult to disentangle the different components of an intervention or to assess interactions between longitudinal activities of the collaborative components. Hence, these encouraging results must be tempered by the limitations of persistent gaps in QIC design, quality of reporting, and publication bias.

### Collaborative learning

The earliest QIC activities and methodology were first described by Northern New England Cardiovascular Disease Study Group, established in 1986 [14], and the Vermont Oxford Network, established in 1988 [15, 16]. This methodology for QIC became popularized as the Breakthrough Series developed by the Institute of Healthcare Improvement in 1995. Many examples in the literature of the QIC initiatives are based on the Breakthrough Series [13], a collaborative improvement model which outlines key elements including multidisciplinary teams working together to improve performance on a chosen topic supported by experts using evidence-based best practices [2]. QI Collaboratives have described improvement in a broad spectrum of topics in adults and children [17–19]. Furthermore, pediatric collaborative improvement networks have demonstrated improved outcomes for children and their families across multiple subspecialities [6, 20].

### QI collaboratives in neonatology

There are several regional, national, and international collaborative networks established in neonatology. These include, but are not limited to, the California Perinatal Quality Care Collaborative (CPQCC), Ohio Perinatal Quality Collaborative (OPQC), Tennessee Initiative for Perinatal Quality Care (TIPQC); Children’s Hospital Neonatal Consortium (CHNC), The Pediatrix Center for Research, Education, Quality and Safety (CREQS), The National Network of Perinatal Quality Collaboratives (NNPQC), the Solutions for Patient Safety (SPS), and Vermont Oxford network (VON); Canadian Evidence-based Practice for Improving Quality (EPIQ) [21], Australia-New Zealand Neonatal Network (AZNN) [22], International Network for Evaluating Outcomes (iNeo) of neonates [23], and Chinese Neonatal Network (CHNN) [24].

In this review, we aim is to evaluate national QI collaboratives primarily based in US (described below in alphabetical order). We will discuss the QI framework used by these collaboratives and provide examples of specific outcomes that their studies addressed.

#### Children’s Hospital Neonatal Consortium

The Children’s Hospital Neonatal Consortium (CHNC) provides a platform in QI for participating children’s hospital neonatal intensive care units (NICUs) by means of Collaborative Initiatives for Quality Improvement (CIQI) [25, 26]. This platform provides leadership, collaborative structure, resource access, QI education, team feedback, data driven change, and several other resources to assist participating institutions in completing QI projects. CIQI Steering Committee is a multi-disciplinary group of QI leaders involving physicians, nurses, and advanced practice clinicians which provides oversight of the CHNC CIQI Program. A CIQI Program Manager is responsible for operations, data management, and coordination of QI activities. At the local level a project planning and management team together with faculty advisors help oversee the collaborative efforts.

#### Children’s Hospitals Solutions for Patient Safety

Launched in 2009, Children’s Hospitals Solutions for Patient Safety (SPS) [27] was founded as a partnership between providers and the business community to improve quality and reduce costs. The mission is to work to eliminate serious patient harm from children’s hospitals by partnering with families and frontline staff. The tenets that are the driving force for SPS include executive leadership, focus on outcomes through HRO concepts and QI methodologies, focus on sharing successes and failures transparently, learning from one another instead of competing, “All Teach, All Learn” principle, and finally a commitment to building a “culture of safety”. SPS aims to decrease rates of the most common serious patient and employee harms, to identify and eliminate safety disparities, and to create robust learning systems for ambulatory safety. The network has now grown to include 140+ children’s hospitals across US and Canada.

#### National Institute for Children’s Health Quality

National Institute for Children’s Health Quality (NICHQ) [28] is committed to achieving better health outcomes for children and their families. It is a mission driven non-profit organization that focuses on providing equitable change for the health of children and their families. The National Network of Perinatal Quality Collaboratives (NNPQC) [29] provides resources and expertise to state-based perinatal quality collaboratives to improve maternal and infant health outcomes, particularly within marginalized populations disproportionately affected by adverse perinatal outcomes.

#### Pediatrix- center for research, education, quality and safety

With the goal to utilize evidence-based medicine to advance clinical care, the Pediatrix Center for Research, Education, Quality, and Safety (CREQS) engages in clinical research, education, continuous quality improvement, and safety initiatives. The specific objectives of CREQS are to contribute to better patient outcomes and reduce long-term healthcare costs. Pediatrix also provides [30, 31] a High Reliability Organization (HRO) program that combines characteristics of HROs with Six Sigma, TeamSTEPPS (Team Strategies and Tools to Enhance Performance and Patient Safety), and Crew Resource Management.

#### Vermont Oxford Network

The Vermont Oxford Network (VON) [32] is a nonprofit voluntary collaboration of health care professionals and families at more than 1400 member hospitals, primarily in the United States, working together as an interdisciplinary community to improve the quality, safety, and value of care for newborn infants and their families. iNICQ is an Internet-Based Newborn Improvement Collaborative for Quality coordinated by VON which brings teams together by using local and collective data to guide improvement on a designated topic. Participating centers are supported with access to expert faculty, VON measurement and reporting, an interactive online toolkit of evidence-based practices, modules, and online classrooms. Newborn Improvement Collaborative for Quality (NICQ) are groups of 8–10 teams that work together creating a culture of collaboration allowing for data-transparency, data-driven and evidence-based approaches, and rigorous quality improvement methodology to achieve and sustain improvement. Each homeroom faculty consists of a QI leader, a family/parent leader, and a NICU clinician. The VON NICQ collaborative includes families as partners to ensure that the family perspective is represented, valued, and enhanced during each improvement cycle.

### Approach and outcomes of QI collaboratives in neonatology

Table 1 shows some examples from each of the 5 collaboratives described. We identified papers that described improvement work within each QIC and did not include individual team reports, or case reports. In addition, we chose a sample of convenience, up to 6 for each QIC that represents different methodologies and various types of clinical issues that QICs have addressed. This sample serves as a representation of the work of each OIC. The number of centers varies based on the size of the collaborative. The number of centers in each of the QIC publications varies from 7 to 330. These collaboratives demonstrate a wide range of topics have been addressed via QIC in neonatology. A clearly defined aim statement was identified for most of these QIC initiatives. However, the operational definitions for outcome, process, and balancing measures have not always been defined in these projects. Although all manuscripts have reported a collective improvement by the collaborative, it is apparent that not all the individual participating centers demonstrate improvement. Some centers contribute more data than others to the outcomes leading to overall improvement in the collaborative. In our evaluation, we identified only one QIC which addressed the economic implications of a QIC, specifically on low-birth-weight infants (NIC/Q- Neonatal Intensive Care Collaborative Quality).

**Table 1:** Examples of QI papers published from each of the 5 collaboratives.

Number	Project title	Topic of interest	Aim	Author/Year	No. sites in study	Summary of outcomes
**Children’s Hospital Neonatal Consortium**						
1	Interdisciplinary Teamwork and the Power of a Quality Improvement Collaborative in Tertiary Neonatal Intensive Care Units [25]	Establish a collaborative infrastructure and reduce central line-associated bloodstream infections (CLABSIs)	Enable completion of meaningful, collaborative QI projects in the CHNC CIQI by achieving targets set on measures	Grover T/2015	17	CLABSI rate decreased from 1.33/1000 line days to a rate of 1.08/1000 line days, a 20% decrease. 11 of the 17 centers showed improvement. The improvement was sustained for 12 months.
2	SLUG Bug: Quality Improvement With Orchestrated Testing Leads to NICU CLABSI Reduction [35]	Reduce central line–associated bloodstream infection (CLABSI)	Decrease collaborative baseline CLABSI rates by a clinically meaningful target of 15% over 12 months	Piazza A/2016	17	14 of the 17 centers had decreased infection rates. Orchestrated testing showed that Hub scrub compliance monitoring in combination with sterile tubing change, had the strongest effect in decreasing CLABSI rates.
3	Sustaining SLUG Bug CLABSI Reduction: Does Sterile Tubing Change Technique Really Work? [36]	Sustain CLABSI rates and assess the impact of the sterile tubing change (TC) technique as a component in CLABSI reduction	(1) report the ability of centers to sustain low rates and (2) describe the impact of the change from clean to sterile TC techniques in the 4 centers over the subsequent months of the sustain phase beginning in January of 2013	Pallotto EK/ 2017	16	The 19.3% collaborative CLABSI rate reduction was sustained for the subsequent 19 months. Four centers adopted the sterile TC technique during the sustain phase and had a 64% fall in CLABSI rates.
4	STEPP IN: Working Together to Keep Infants Warm in the Perioperative Period [55]	Collaboration between Neonatology and Anesthesia for perioperative temperature management in Neonates	Decrease the incidence of hypothermia by 50%, from a baseline of ∼20% to 10%, by December 2014 and sustain over 12 months.	Brozanski/2020	19	Postoperative hypothermia decreased by 48%, from a baseline of 20.3% to 10.5% .
5	STEPP IN: A Multicenter Quality Improvement Collaborative Standardizing Postoperative Handoffs [56]	Collaboration between Neonatology and Anesthesia for postoperative communication	Handoff improvement to reduce care failures by 30% and implement a standardized communication process for postoperative handoff.	Piazza A/2021	19	Communication failures specific to respiratory care decreased by 73.2%. All other communication care failures decreased by 49.4%.
6	A Multicenter Collaborative to Improve Postoperative Pain Management in the NICU [53]	Decrease postoperative pain and improve family satisfaction with pain management	Decrease the percentage of patients with unrelieved postoperative pain from 19.5% to 15% or less and improve family satisfaction with pain management to ≥90% in the first 24-hours postop. Sustain the improvement for 6 months	Bapat R/2023	23	The percentage of patients with unrelieved pain decreased by 35% from 19.5% to 12.6%. Family satisfaction with pain management increased from 93% to 96%. Improvements maintained during sustain period.
**Children's Hospitals Solutions for Patient Safety**						
1	Children’s Hospitals’ Solutions for Patient Safety Collaborative Impact on Hospital-Acquired Harm [57]	To determine if reliable best practice implementation and culture of safety improvements can reduce hospital-acquired conditions (HACs) and serious safety events (SSEs).	The team for each of the 9 HACs developed their own specific aim along with a target of 90% compliance to the bundles.	Lyren, A/2017	32 sites for HACs and 21 for SSEs	Significant harm reduction occurred in 8 of 9 common HACs (range 9%–71%; *P* < 0.005 for all). The mean monthly SSE rate decreased 32% (from 0.77 to 0.52; *P* < 0.001).
2	Impact of a Pressure Injury Prevention Bundle in the Solutions for Patient Safety Network [58]	To describe changes in pressure injury (PI) rates in pediatric hospitals after implementation of an active surveillance and prevention bundle and to assess the impact of bundle elements.	To reduce the number of serious PI defined as stage 3, stage 4, unstageable pressure injuries, and deep tissue injuries	Frank, G/2017	33	The rate of stage 3 PI declined from 0.06 to 0.03 (*P* < 0.001), stage 4 pressure injuries declined from 0.01 to 0.004 per 1000 patient-days (*P* = 0.02). The cohort that achieved 80% prevention bundle compliance had significantly lower PI rates.
3	Assessment of an Unplanned Extubation Bundle to Reduce Unplanned Extubations in Critically Ill Neonates, Infants, and Children [59]	To determine if a QIC initiative targeting all intubated neonatal and pediatric patients is associated with a reduction in UEs and morbidity associated with UE events.	To reduce the absolute rate of UEs to < 1UE/100 ventilator days over a 2-year period	Klugman, D/2020	43	Aggregate reduction in UE events by 24.1%, from a baseline rate of 1.135 UEs to 0.862 UEs per 100 ventilator days. Pediatric ICU showed an absolute reduction in UE events of 20.6% and neonatal ICU demonstrated 17.6% reduction.
4	The Relationship between High-reliability practice and Hospital-acquired conditions among the SPS Collaborative [60]	Evaluating the association between integrating high-reliability practices and patient harms to inform a patient safety strategy across the healthcare landscape	To evaluate the association between high-reliability practices and hospital-acquired conditions	Randall, K/ 2021	25	In this nonexperimental design study, there was a significant inverse relationship between the culture of safety component score and the Serious Harm Index (*p* < 0.03) indicating that integration of high-reliability principles may support improved patient safety.
5	Association Between Hospital-Acquired Harm Outcomes and Membership in a National Patient Safety Collaborative [61]	Understand the true effect of large-scale, government-funded collaborative improvement programs to guide policy and practice around health care improvement.	To evaluate associations between membership in SPS and hospital-acquired harm using standardized definitions and secular trend adjustment.	Coffey M/ 2022	99	Comparing early adopters to late adopters, implementation of the SPS was associated with an improvement in HAC rates in 3 of the 8 conditions: central catheter–associated bloodstream infections, falls of moderate or greater severity, and adverse drug events.
6	Pediatric Ventilator-Associated Events Before and After a Multicenter Quality Improvement Initiative [62]	To assess whether adherence to 1 or more test factors in a QI bundle would reduce PedVAE rates.	Decrease the PedVAE rate by 20% by December 2020	Wu AG/2023	95	PedVAEs prevention QI bundle decreased the rate by 26% in hospitals that received training and education. In hospitals that did not implement such interventions, improvement was not noted.
**National Institute for Children's Health Quality**						
1	Lessons Learned from Hospital Leaders Who Participated in a National Effort to Improve Maternity Care Practices and Breastfeeding [63]	A national QI collaborative of hospital leaders designed to accelerate the number of Baby-Friendly–designated hospitals focused on maternity care practices and breastfeeding.	To have an additional 90 hospitals in the United States designated as Baby-Friendly	Feldman-Winter L/ 2016	89	Leadership QI training served as a vital catalyst resulting in 89 newly designated Baby-Friendly hospitals.
2	Best Fed Beginnings: A Nationwide Quality Improvement Initiative to Increase Breastfeeding [64]	To increase breastfeeding and achieve Baby-Friendly designation	By September 30, 2014, 100% of the participating hospitals are designated as Baby-Friendly or have a BFUSA site visit scheduled	Feldman-Winter L/ 2017	90	80% of hospitals received the Baby-Friendly designation. Breastfeeding increased from 79% to 83%, and exclusive breastfeeding increased from 39% to 61%.
3	Maternity Care Clinicians’ Experiences Promoting Infant Safe Sleep and Breastfeeding During the COVID-19 Pandemic [41]	Clinicians’ perceptions and experiences of promoting infant safe sleep (ISS) and breastfeeding during the COVID-19 pandemic	No prespecific aim mentioned	Menon M/ 2023	10	Descriptive qualitative study of 29 clinicians from 10 hospital teams. Key informant interviews identifies 4 main themes: Strain on Clinicians Related to Hospital Policies, Coordination, and Capacity; Effects of Isolation for Parents in Labor and Delivery; Reevaluating Outpatient Follow-Up Care and Support; Adopting Shared Decision-Making.
**Pediatrix- Center for Research, Education, Quality and Safety**						
1	Improving Growth of Very Low Birth Weight Infants in the First 28 Days [65]	Improving neonatal growth	To increase weight gain in the first 28 days after birth for very low birth weight (VLBW) infants	Bloom BT/ 2003	51	Average daily weight gain increased from 10.4 ± 6 g to 12.5 ± 6 g. 76% units noted improvement.
2	Comprehensive Oxygen Management for the Prevention of Retinopathy of Prematurity: The Pediatrix Experience [66]	Development of Electronic Health Records, tools for QI initiatives and examples of QI initiatives (COMP-ROP (Comprehensive Management of ROP)	The COMP-ROP Collaborative- no prespecific aim mentioned	Ellsbury D/ 2010	80	A decrease in severe ROP (stage 3, 4, 5, or surgical) in infants with birth weights of 400 to 1500 g from 11% to 5.8%.
3	A Multifaceted Approach to Improving Outcomes in the NICU: The Pediatrix 100 000 Babies Campaign [67]	Generate large-scale simultaneous improvements in multiple domains of care in a large neonatal network.	Improve performance in targeted process and outcome measures by 10% by 2013	Ellsbury D/ 2015	330	Human milk feeding, exposure to medications that were targeted for reduction (dexamethasone, H2 blockers, metoclopramide, and cefotaxime), ventilator days, admission temperature all improved (*p* < 0.0001). Mortality, necrotizing enterocolitis, retinopaty of prematurity, late onset sepsis, and CLABSI all decreased. Survival without significant morbidity improved.
**Vermont Oxford Network**						
1	Collaborative Quality Improvement for Neonatal Intensive Care [16]	To make measurable improvements in infection and chronic lung disease outcomes using a multidisciplinary QIC model.	Reduction in the nosocomial infection rate for infants 501 to 1500 g to the 25th percentile and absolute reduction in the rate of death or oxygen supplementation at 36 weeks’ postconceptional age by 10% for infants 501 to 1000 g with gestational ages < 34 weeks.	Horbar, J/ 2001	10	The rate of infection with coagulase-negative staphylococcus decreased from 22.0% to 16.6% (*p* = 0.007)and death or supplemental oxygen at 36 weeks’ adjusted gestational age decreased from 55.9% to 47.6% (*p* = 0.039). There was heterogeneity in the effects among the NICUs in both project groups.
2	Economic Implications of Neonatal Intensive Care Unit Collaborative Quality Improvement (NIC/Q) [50]	To describe the economic implications of a collaborative QI effort for very low birth weight infants in the NIC/Q.	Data on treatment costs and data on resources were collected.	Rogowski, J/2001	10	The median treatment cost per infant with birth weight 501 to 1500 g in the infection group decreased from $57,606 to $46,674 (*p* < 0.0001); at the 4 chronic lung disease hospitals decreased from $85,959 to $77,250 (*p* = 0.7980).
3	Improving Care for Neonatal Abstinence Syndrome (NAS) [68]	To determine if the collaborative was effective in standardizing hospital policies and improving patient outcomes for infants with NAS.	A multicenter, multistate QIC focused on infants requiring pharmacologic treatment for NAS.	Patrick, S/ 2016	199	NAS focused guidelines increased. The median length of pharmacologic treatment decreased from 16 days to 15 days (*P* = 0.02), and LOS from 21 days to 19 days (*P* = 0.002). Fewer babies, 39.7% vs. 26.5%, wnet home of medication (*p* = 0.02)
4	Alarm safety and oxygen saturation targets in the VON iNICQ 2015 collaborative [69]	Prospective multicenter audits assessed implementation of policies addressing Joint Commission 2014 Alarm Safety goals	To assess progress in VON iNICQ 2015: Alarm Safety Collaborative in achieving Joint Commission 2014 alarm safety goals with respect to oximeters, and to compare patient-level oxygen saturation (SpO2) and oximeter alarm data to local policies.	Hagadorn, JI/ 2017	86	Of 13 policies addressing mandated goals, 8 policies were implemented at audit 1 and 9 at audit 2 (*P* = 0.004). At audit 1, 28 NICUs had implemented ⩾ 9 policies versus 47 at audit 2. Median SpO2 target lower limit was 88% (interquartile range 87%, 90%; range 75%–94%), upper limit 95% (interquartile range 94%, 96%; range 85%–100%).
5	A Collaborative Multicenter QI Initiative to Improve Antibiotic Stewardship in Newborns [70]	Achieve increased compliance with the Centers for Disease Control and Prevention (CDC) core elements for antibiotic stewardship and demonstrate reductions in antibiotic use (AU) among newborns	To assess the progress of the participating NICUs with respect to achieving the CDC core elements of antibiotic stewardship and measure the AUR over the 2 years of this collaborative. Individual teams were encouraged to develop SMART aim statements	Dukhovny D/2019	146	The percentage of NICUs implementing the CDC core elements increased in each of the 7 domains. The median AU rate decreased from 16.7% to 12.1% (*p* < 0.0013), a 34% relative risk reduction.
6	Implementing an exclusive human milk diet for preterm infants: real-world experience in diverse NICUs [71]	Human milk–based human milk fortifer (HMB-HMF) makes it possible to provide an exclusive human milk diet (EHMD) to very low birth weight (VLBW) infants in NICU.	Increase the utilization of an EHMD in the NICU population	Swanson, J/2023	7	EHMD programs were cost effective. EHMD programs resulted in either a decrease or change in total (medical+surgical) NEC rate and reductions in surgical NEC. Institutions that provided cost and complications data reported a substantial cost avoidance after EHMD implementation, ranging between $515,113 and $3,369,515 annually per institution.

The implementation and mechanism of impact of a QIC varies widely and are briefly summarized here. CHNC utilizes the Model for improvement [33] methodology in their QI efforts. The collaborative provides best practices through clinical practice recommendations, but each center develops their own tools to conduct the project. IHI Breakthrough Series Collaborative Framework [13] concepts are used through regularly scheduled monthly meetings and huddles which provide opportunity to educate, share challenges, find solutions, report progress and data. Shared learning helps each center achieve their goals. CHNC also successfully utilized orchestrated testing [34], an application of planned experimentation that allows simultaneous examination of multiple practices (bundle elements) to determine which intervention or combination of interventions are the most effective. This technique has been used to reduce central line–associated bloodstream infections [35, 36].

100,000 Babies Campaign, first launched by Pediatrix in 2007, utilizes a QI infrastructure based on the Kotter organizational change model [37, 38]. QI projects in this collaborative utilize electronic health records- Pediatrix BabySteps Clinical Data Warehouse which provides a valuable resource for population and outcomes data [39]. The 100,000 Babies Campaign used QualitySteps [39, 40], a system developed by Pediatrix to assist development of project design, worksheets, and annotated run charts for CQI projects. This program continues to provide QI opportunities through its version 2.0.

NICHQ developed a change package and provided technical assistance to incorporate QI principles to improve the systems of care for premature babies during the Neonatal Outcomes Improvement Projects (2007–2010) in several states to reduce the burden of mortality and morbidity associated with premature birth and low birth weight. Some of the neonatal related initiatives by the organization includes but not limited to Best Fed Beginnings [41], a national QI initiative to help increase the number of “Baby-Friendly” designated hospitals in the United States; Infant Mortality Collaborative Improvement and Innovation Network, safe sleep, smoking cessation, social determinants of health, perinatal regionalization, and NewSTEPs 360, a national collaboration that aims to improve newborn screening programs.

The SPS network utilizes QI Science & HRO Concepts to drive the culture to reduce harm. SPS has developed change packages that inform best practices to reduce Hospital Acquired Conditions (HACs) such as Central line associated blood stream infections, unplanned extubations, adverse drug events, pressure injuries, and others. The change packages also include measurement strategy, care bundles, toolkits, and action items to inform improvement. Another key element SPS incorporates is partnering with and engaging patients and families in their QI initiatives.

VON utilizes the Model for Improvement [33] and experience-based co-design in their QI efforts. VON develops toolkits that consist of change ideas and evidence based potential better practices that are available to the teams. The term “potentially better practices” indicates that a practice is not considered better or best until adapted, tested, and shown to work in the local context. VON QI collaborative provides resources including clinical examples, measurement plans and reporting, foundational QI resources, consultation and collaboration with care experts and other teams. Teams test and implement evidence-based changes and apply continuous measurement to demonstrate improved outcomes. The collaborative uses the approach Learn + Measure + Share = Improve. Online courses related to QI foundations, VON Grand rounds and webinars provide evidence-based education to participating centers.

## Discussion

In this review, we have described the framework and initiatives of 5 neonatal QICs that have demonstrated improved outcomes for neonates. These QICs are part of a broader network of collaborative efforts throughout the USA. At the grassroots level, the collaboratives offer a systematic approach towards improvement work, identification of common specific aims, and a standardized approach towards data collection, data analysis, benchmarking, reporting of results, and sharing best practices. Collaboratives are designed to have a broader impact across multiple centers by promoting collective learning. Our review indicates that the strategies of implementation of interventions vary with each QIC initiative. The most common approach is to develop ‘best practice’ focused interventions or bundle elements allowing centers to choose the interventions that are best suited for their local context. Other QICs offer specific interventions which centers must implement. Examples of more specific planned interventions include orchestrated testing, which was used to reduce central line–associated bloodstream infections. The perceived strength of a QIC is the collaboration of a group of experts and committed individuals in improving an outcome, a structured approach, sharing of best practices, collective learning, undertaking rapid cycles of change thereby leading to improvement [6, 42, 43].

Multiple factors contribute to the success of a QIC. At the QIC level, a structured framework, fostering a collaborative environment, data abstraction and data access, transparent sharing of data and interventions, identification of outcomes with clear operational definitions, effective use of technology, utilizing sound QI methodology, engagement of multidisciplinary stakeholders and families are paramount. In our review, we have identified that improvement is not uniform across all centers. At the individual institution level, participating smaller institutions lacking resources can perform studies using resources provided by the collaborative. Additionally, irrespective of whether an initiative is at individual center or part of a collaborative, at the individual center level, addressing local barriers, local leadership, organizational factors, team dynamics plays an important role [42]. One of the strengths of a QIC is that it enables less resourced hospitals to perform effective quality improvement initiatives.

Provider roles and strategies in single-center QI projects vs QICs has not been clearly described in the literature [44]. Single-center QI projects are often designed within the scope of an institution’s requirements, policies, and procedures. In single-center QI projects, since the improvement team is aware of the context, the interventions are targeted towards the local context. Since micro/macro environments and resources differ amongst every healthcare institution, one might argue that the lessons learned may not be generalizable in other settings.

On the other hand, QICs include multiple centers with different systems and resources. QICs offer a benefit over individual QI initiatives since the improvement work has been conducted in multiple different contexts and on a larger patient population. Hence, the interventions and results in a QIC may be more applicable in multiple settings as opposed to single institution QI initiatives. If nonparticipating individual institutions want to incorporate a similar improvement in their own healthcare system, the strategies from a QIC may be more adaptable. Additionally, challenges to sustaining improvement, to spreading change, and identifying stable funding sources exist in QIC much like single-center QI.

QICs have limitations that should be considered as areas of opportunity for further work. While all the QIC publications describe a positive outcome, there were institutions that participated in the collaboratives that did not show improvement. The individual contributions of each participating center towards the collaborative outcome varies but this variability is not always taken into consideration in QIC analysis. Perhaps, when reporting the QIC results, recognition can be made of the unequal contributions of the individual institutions. Furthermore, a detailed documentation of local processes is beneficial [44] to compare the outcomes and adaptability of QIC results so that readers can decide what approaches to incorporate in their own context. In addition, there could be publication bias, with positive studies being published more often, which may influence the conclusions one draws from a QIC [9, 45–47]. A study by Olson and colleagues [48] suggests that journal editors may not have a strong bias towards publishing positive results once manuscripts are submitted, but rather that researchers are more inclined to submit studies with positive results. We suggest that prospective registration followed by publication of the QIC efforts irrespective of whether the QIC had positive or negative results will help with accurate reporting of the effectiveness of QICs [47]. Although most QICs report on outcomes, further improvement work related to cost effectiveness and value [49, 50], evaluating the data and targeted interventions of a QIC from an equity lens [51, 52], and engaging families [53] are important next steps. As described in a recent commentary, local change, QIC, and equity focused QI are all necessary strategies to improve outcomes [54]. In relation to academic advancement, in QICs oftentimes the local project leader may not receive credit for the publication while the credit goes to the QIC management team. This factor may deter some from joining a QIC which could introduce a sampling bias. Our review is limited since we did not perform a comprehensive review of all collaborative neonatal QI but rather selected examples to illustrate their process and outcomes. Lastly, by limiting this review to collaboratives primarily based in the United States, some methods used in other countries may have been excluded.

## Conclusions

QICs play a significant role in identification of target topics, developing best practices, improving provider knowledge, building QI capacity, and improving outcomes in neonates. QICs offer a benefit over individual QI initiatives since the improvement work has been conducted in multiple contexts. QICs are valuable since the knowledge gained is more generalizable and adaptable. Simply by enrolling, a center is not guaranteed improvement. In the description of the QICs, the individual contributions of each participating center towards the collaborative outcome varies. Although the results from the QICs we chose for this review are quite promising, these encouraging results must be tempered by the limitations of persistent gaps in QIC design, quality of reporting, and publication bias. Finally, the knowledge gained from our qualitative synthesis is valuable and could be taken into consideration for strategic planning of future QICs.
